# Does Exercise-Based Conventional Training Improve Reactive Balance Control among People with Chronic Stroke?

**DOI:** 10.3390/brainsci11010002

**Published:** 2020-12-22

**Authors:** Lakshmi Kannan, Jinal Vora, Gonzalo Varas-Diaz, Tanvi Bhatt, Susan Hughes

**Affiliations:** 1 Department of Physical Therapy, University of Illinois at Chicago, Chicago, IL 60612, USA; lkanna2@uic.edu (L.K.); vorajinalp11@gmail.com (J.V.); gvaras2@uic.edu (G.V.-D.); 2 College of Applied Health Sciences, University of Illinois at Chicago, Chicago, IL 60612, USA; 3 Department of Community Health Sciences, School of Public Health, University of Illinois at Chicago, Chicago, IL 60608, USA; shughes@uic.edu

**Keywords:** chronic stroke, conventional therapy, exercises, reactive balance control

## Abstract

Background: Exercise-based conventional training has predominantly benefited fall-associated volitional balance control domain; however, the effect on reactive balance control is under-examined. Therefore, the purpose of this study was to examine the effect of exercise-based conventional training on reactive balance control. Methods: Eleven people with chronic stroke (PwCS) underwent multi-component training for six weeks (20 sessions) in a tapering manner. Training focused on four constructs-stretching, functional strengthening, balance, and endurance. Volitional balance was measured via movement velocity on the Limits of Stability (LOS) test and reactive balance via center of mass (COM) state stability on the Stance Perturbation Test (SPT). Additionally, behavioral outcomes (fall incidence and/or number of steps taken) were recorded. Results: Movement velocity significantly increased on the LOS test (*p* < 0.05) post-intervention with a significant decrease in fall incidence (*p* < 0.05). However, no significant changes were observed in the COM state stability, fall incidence and number of recovery steps on the SPT post-intervention. Conclusion: Although volitional and reactive balance control may share some neurophysiological and biomechanical components, training based on volitional movements might not significantly improve reactive balance control for recovery from large-magnitude perturbations due to its task-specificity.

## 1. Introduction

Stroke is the leading cause of long-term disability, and more than half of the community-dwelling people with stroke live with residual sensorimotor and balance impairments [1]. Due to such impairments, approximately 40–70% of them experience a detrimental fall each year in the United States, creating significant healthcare costs [2,3,4,5,6,7,8,9]. Remaining physically active after stroke is essential for recovery, maintaining quality of life, and reduces risk of secondary stroke [10]. However, impaired balance control has been associated with high incidence of falls and reduced walking abilities, thereby increasing the fear of falling and affecting community participation [11,12].

Balance control involve volitional and reactive domains which are activated by the central nervous system in response to self-induced or an externally induced perturbation, respectively [13]. Voluntary adjustments are associated with feedforward mechanisms which contribute to planning and executing movements by directing the body segments towards an intended goal and simultaneously maintain balance control against self-induced perturbations [13,14,15,16]. On the other hand, reactive postural adjustments via compensatory stepping use feedback mechanisms to prevent balance loss against a sudden external perturbation [17]. There are several reliable and valid tools to assess these balance control domains and fall-risk among people with chronic stroke (PwCS). Volitional balance control is usually assessed via measuring performance on self-initiated functional tasks in clinical balance tests (i.e., Berg Balance Scale, Functional Reach Test, Timed Up-and-Go, Limits of Stability test (LOS), etc.). Reactive balance control is assessed by measuring postural stability and the compensatory in-place or stepping responses in response to externally induced perturbations via platform or treadmill translations [18,19,20,21,22].

Several review studies have described that volitional balance control can be significantly improved via exercise-based conventional training (EBCT) methods focusing on balance, gait, functional weight shifts, strength and agility in PwCS [23,24,25,26,27,28,29,30,31]. Additionally, some evidence indicates that alternative therapies such as Yoga, Tai-chi, dance-based exergaming are also effective in reducing fall risk in PwCS. These interventions are classified as task-specific based on the characteristics of the training which include activities of daily living such as sit-to-stand transfers, standing balance with reach outs, and treadmill gait training [23,24,25,26,27,28,29,30,31]. In this regard, the training-induced gains within the volitional balance control domain could be attributed to the task-specific nature of EBCT methods in which the exercises target self-initiated activities in response to explicit instructions or anticipated conditions [23,24,25,26,27,28,29,30,31,32]. However, evidence on generalization or the extent to which such training paradigms improve reactive balance control remains unclear.

Specifically designed volitional training consisting of agility and step training along with other balance exercises have shown beneficial effects in improving reactive balance control outcomes. Marigold et al. [33] demonstrated a significant decrease in muscle onset latencies and step reaction times to platform translations in PwCS after undergoing agility training compared to weight-shift training group [33]. Similarly, Vearrier et al. [34] demonstrated a significantly reduced time to stabilization of the center of pressure in response to platform translations after an intensive static and dynamic functional balance tasks majorly addressing strength and range of motion [34]. Despite that such exercises were volitionally driven, improvements within the reactive balance control domain could be postulated to occur due to the partial sharing of neural resources between volitional and reactive control domains [35,36]. It has been suggested that while there is a temporal distinction observed in muscle synergy patterns across voluntary and reactive motor behaviors, there are similarities in spatial recruitment of muscle synergy patterns between both voluntary and reactive motor responses [37,38,39,40,41]. Additionally, it has been demonstrated that only the temporal aspects of muscle synergy patterns are distorted post-stroke, leaving the spatial patterns unaltered [42,43]. Further from a neurophysiological perspective, while it is postulated that the initial reactive response to external-induced perturbations might be more “automatic” and mediated by spinal short-latency and brainstem long-latency responses, the later parts of the cortical reactive response involve cortical modulation, especially to large-scale perturbations [13,18]. Considering volitional exercises mediated via cortical control there might be some potential overlap between the control mechanisms indicating a possibility of volitional training to generalize to reactive stepping. Such neurophysiological evidence could support the idea that EBCT, including task-specific volitional exercises, could enhance reactive balance behavior and reduce fall incidence which may be more feasible and easy to implement [33,34]. Lastly, a recent study demonstrated that volitional gait stability and hip extensor strength are significant predictors for slip-induced fall-risk [44], which confirm the relation between voluntary movements and reactive responses. Hence, EBCT targeting balance, gait and strength training could improve fall-risk from external perturbations.

Further several systemic reviews and meta analyses aimed to investigate the effect of multi-component therapeutic intervention on balance control have reported promising effects on reducing fall risk in older adults and PwCS [25,31]. Although both volitional and reactive balance control domains might have contributed towards the fall-risk reduction reported, most of these studies in PwCS have only assessed outcomes within the volitional balance control domain [23,24,25,26,27,28,29,30,31]. The few studies that have examined reactive outcome have used smaller magnitudes of perturbation intensities and might not represent realistic environmental perturbations and might not have been sufficient to induce a balance loss–often a precursor to falls [45,46]. Additionally, there exists a discrepancy in the manner EBCT is designed resulting in variability of results in reducing fall risk thereby warranting the need to design effective intervention components. Among this line, evidence regarding the effect of EBCT on reactive balance components remains limited and therefore needs to be studied for designing effective therapeutic strategies to prevent falls in PwCS.

To the best of our knowledge, no study has examined the extent to which a customized exercise-based conventional training (EBCT) intervention including the domains of stretching, strengthening, balance control and proprioception, and endurance can benefit compensatory strategies against large magnitude perturbations in PwCS. The primary aim of this pilot study was to examine the effect of a six-week, multi-component, EBCT on both reactive (Stance Perturbation test, SPT) and volitional (Limits of Stability test, LOS) balance domains in PwCS. We hypothesized that the EBCT protocol would result in fall-risk reduction from improvements in both reactive and volitional balance control and fall-risk, with higher improvements throughout volitional measures of stability and clinical balance tests compared to reactive variables of balance control.

## 2. Materials and Methods

### 2.1. Methods

#### 2.1.1. Participants

The study was approved by the University of Illinois at Chicago institutional review board (2012-0111). Twenty community-dwelling people with self-reported hemiparetic chronic stroke (>6 months) were included in the study after obtaining informed consent. Post-screening a total of 13 participants were included in the study, of which only 11 people (60.63 ± 4.24 years of age) completed the study. These participants were recruited by posting flyers at University of Illinois hospital, stroke support groups, outpatient rehabilitation clinics and research centers.

#### 2.1.2. Participants’ Eligibility

Individuals with chronic hemiparetic cortical stroke (onset >6 months) without any presence of aphasia, as confirmed by their physician, were included. They were required to have the ability to stand independently for at least 5 min without the use of an assistive device or physical assistance and needed to follow instructions in English. Heel bone density scan was measured for these individuals using the Lunar Achilles Insight, and a T-score of less than −2.0 were excluded as they were classified as osteopenia or osteoporotic. Individuals were excluded if they self-reported a history of musculoskeletal, cardiac, or neurological impairment other than stroke, or the presence of metal implants due to any of the above-mentioned conditions.

### 2.2. Protocol

#### 2.2.1. Intervention

Included participants underwent 6 weeks of multi-component, exercise-based conventional training (EBCT) in a tapering format [47] (i.e., 5 sessions/week for 1st and 2nd weeks, 3 sessions/week for 3rd and 4th weeks, and 2 sessions/week for 5th and 6th weeks) for a total of 20 sessions lasting 90 min each. The custom-designed training protocol primarily focused on 4 main constructs: stretching, strengthening, balance and proprioception, and cardiovascular endurance. Each session began with a 10-min warm-up that included self-stretching exercises (trunk twists, trunk flexion and extension, upper limb stretching with active movements, forward and side lunges). Strengthening lasted 20 min and included upper and lower limb exercises with dumbbells and therabands (shoulder/elbow/hip/knee flexion and extension, hip abduction and adduction, high-stepping, squats, and forward and side lunges with/without holds). Balance and proprioception exercises (20–25 min) comprised sit-to-stands, one leg standing, stepping over stools of different heights using both the paretic and non-paretic limbs, foam or rocker bottom standing, sideways walking, postural training on a therapeutic ball with forward and side reaches, standing with eyes closed, and tandem standing which progressed to tandem walking. These exercises focused on stepping activities thereby promoting the ability to weight-bear on paretic as well as non-paretic leg simultaneously improving joint proprioception. Lastly, to address cardiovascular endurance, exercises were followed by treadmill walking for 10–15 min at a self-selected speed that was gradually increased. For progression, figure-of-eight walking, obstacle crossing, and stair climbing were included in endurance training. While the EBCT protocol was structured to address general balance impairments, exercise parameters were tailored to provide a comparable level of challenge for everyone. Each session was face to face and individualized.

#### 2.2.2. Outcome Measures

At baseline (week 0) and post-training (week 7), participants performed instrumented balance tests to assess reactive balance control (SPT) and volitional balance control (LOS). In addition, standardized functional balance outcome measures such as the Berg Balance Scale, Timed Up-and-Go test, Four Square Step Test, 6-Minute Walk Test, and Chair Stand Test were also examined.

Reactive Balance Control: The SPT test was administered using the Active step (Simbex, Lebanon, NH) motorized treadmill. An eight-camera motion capture system with a sampling rate of 120 Hz was used to record full body kinematics (Motion Analysis, Corporation, Santa Rosa, CA, USA) using Cortex software (Motion Analysis powered by Cortex, 6085 State Farm Drive Suite 100, Rohnert Park, CA, USA). Participants were secured in a safety harness attached to an overhead metal arch. Helen Hayes marker set with 29 markers was placed bilaterally on bony landmarks. The head and trunk were used to compute joint centers and the center of mass (COM) [48]. An additional marker was placed on the treadmill to identify the instant of perturbation onset. A load cell was connected in series with the harness in order to measure the amount of body weight exerted on the harness. Participants were asked to attain a comfortable stance position with their feet shoulder width apart. They were instructed that the belt on which they were standing may suddenly move forward or backward and they were asked to respond naturally to regain their balance by taking a step. A familiarization trial was provided before the actual test without any cue given before perturbation onset. The belt moved forward (slip-like) or backward (trip-like) at acceleration of 0.67 m/s for 0.041 s with an acceleration of 16.75 m/s^2^ at a distance of 0.019 m. Kinematic variables, such as the COM, was computed using a custom written algorithm in MATLAB version 2014b (The MathWorks Inc., Nactick, MA, USA) [49].

Postural COM state stability: For the SPT, the COM state stability was computed by examining the control of horizontal COM position and velocity in the anteroposterior direction relative to the base of support (BOS) and expressed about the rear edge of BOS (rear heel) [50]. The COM position was normalized to each individual’s foot length and COM velocity was normalized by the factor *√gxbh*, where *g* is the gravitational acceleration and *bh* is the individual’s body height [51,52]. Stability was calculated at heel touchdown of the compensatory step and represents the shortest perpendicular distance from the instantaneous COM motion state to the theoretical dynamic feasible boundary against backward (slip) or forward (trip) loss of balance [52,53,54]. While greater stability values for slip conditions indicated greater stability, greater stability values for trip conditions indicated greater instability.

Volitional Balance Control: The balance master instrument was used to administer the LOS test [55]. Participants were secured in a safety harness where they stood with their feet on a force platform with a screen in front which provided visual feedback concerning their movement. They were instructed to lean their body in either forward or backward directions upon hearing a beep without stepping or raising their heels off the platform, losing their balance, or holding onto the harness. The platform recorded the center of pressure (COP) excursion. A familiarization trial was provided, after which the actual data was collected.

Movement Velocity (MVL): Volitional balance control task was quantified by recording movement velocity (MVL) which is degrees of movement/second of the movement in either forward or backward direction. Higher values of MVL indicates better performance and has been associated with predicting falls and fall efficacy [55]. Additionally, MVL is known to be a sensitive and reliable outcome for determining volitional balance control impairment [56,57,58].

Fall: For the SPT, the trial was identified as a fall if the participant failed to initiate a step, resulting in a catch by the harness and/or if the force exerted on the load cell exceeded 30% of the participant’s body weight for more than one second after perturbation onset [59]. A fall outcome on the LOS test was identified by the Equitest (through their custom-designed algorithm) and was verified manually (subjects leaning in the harness and needing external assistance to recover).

### 2.3. Clinical Balance Tests

Berg Balance Scale (BBS) measures static stance and dynamic balance while performing moving activities, and is known to predict the risk of falls [60,61]. The 14 items in the test has a maximum score of four on each item. The total score obtained (out of 56) was recorded.

Timed Up-and-Go test (TUG) assesses balance and mobility during walking and has shown to have high reliability for people with stroke [62]. Participants were instructed to get up from the chair, walk three meters, and walk back and sit back down in the chair. The time (seconds) taken to perform the test was recorded.

Four Step Square Test (FSST) measures dynamic balance during standing, particularly the ability to step rapidly in different directions and avoid an obstacle which is essential to avert falls [63]. The participant stepped over four walking sticks in clockwise and anti-clockwise directions, eliciting a step sideways, backwards, and forwards. The time (seconds) taken to complete the sequence was recorded.

Six-Minute Walk Test (6MWT) measures aerobic capacity and is also reflective of community mobility among community-dwelling PwCS [64]. Participants were instructed to walk on a 30 m walkway to cover as much distance as possible within six minutes. The total distance walked was recorded.

Lateral step test (LST) measures strength of the lower extremities (nonparetic and paretic), particularly the coordinated muscle activity of the knee and hip extensors, the hip adductors, and the ankle plantar flexors [65]. Participants were instructed to stand facing the wall with the testing leg on the ground and the other leg placed on a 7 cm high step [65]. They were instructed to step up and down with the non-test leg and number of steps completed within 15 s were recorded [65].

30 s Chair Stand Test (CST) measures the functional strength in lower limb muscles [66]. Participants were asked to cross their arms across their chest and instructed to stand-up and sit back down in a chair as many times as possible within 30 s. The total number of stand-ups were recorded.

### 2.4. Statistical Analysis

Descriptive measures are summarized as means ± SDs or as percentages. The analysis was performed using SPSS (SPSS Inc., version 22, Chicago, IL, USA). Paired t-tests were used to compare differences between pre-to post-intervention for reactive balance control due to the difference in the meaning of values under slip and trip conditions. A two by two ANOVA was performed for the LOS test variables to determine the effect of intervention on volitional balance control. The effect size (d’) was computed using the formula d’ = d/s_d_ where d is the mean difference in scores and s_d_ is the standard deviation of the difference in scores. The effect size index was then defined using Cohen’s classification of the effect size index (d). Values of 0.20, 0.50, and 0.80 indicate small, medium, and large effect sizes, respectively [67]. A linear regression analysis was performed to determine the relationship between laboratory measures (slip stability, trip stability, MVL during backward, MVL during backward) and clinical measures (BBS score, time to complete TUG, time to complete FSST, distance covered in 6MWT, number of steps in LST and number of sit-stands in CST). To do this, delta (improvement or decrement pre to post training) was computed for all laboratory and clinical measures. Adjusted R squared values for the model fit and beta values for the influence of each predictor was determined. Alpha level was set at *p* < 0.05.

## 3. Results

The study participants had a mean age of 60 years with a near equal distribution of gender and stroke side. Participants had an average of 10 years since onset of stroke and more hemorrhagic than ischemic strokes. Participants also demonstrated leg and foot impairment as demonstrated by the Chedoke–McMaster stroke assessment. The details of demographic characteristics of participants are presented in Table 1.

### 3.1. Balance Control Domains

#### 3.1.1. Reactive Balance Control

**Figure 1:**
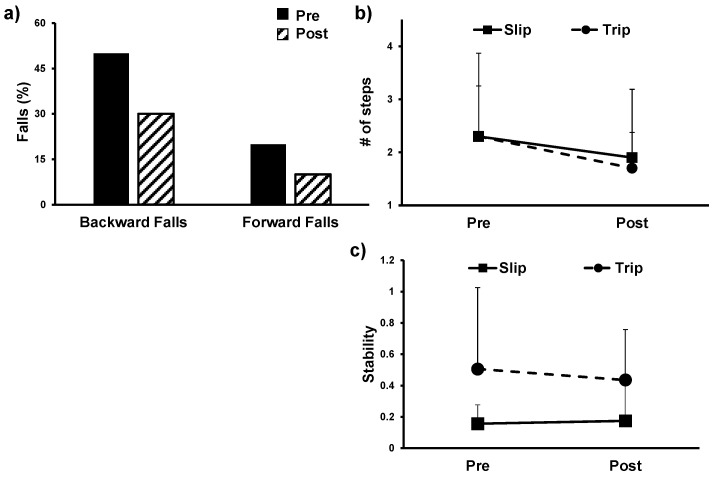
(**a**) display change in fall percentage pre- to post-intervention on Stance Perturbation Test (reactive balance control) in backward and forward direction (**b**) Change in number of compensatory steps pre- to post-training during both slip- and trip-like perturbations (**c**) Change in COM state stability in the stance perturbation test during both slip- and trip-like perturbations.

Paired *t*-test revealed no significant improvement in the COM state stability (*t*(9) = −0.21, *p* > 0.1) pre- to post-training under slip conditions with a very small effect size (0.09). Similarly, there was no significant improvement in COM state stability (*t*(9) = 0.55, *p* > 0.1) pre- to post-training under trip conditions with a small to medium effect size (0.4). Although the number of falls decreased from 45% to 27% during slip conditions (*t*(10) = −1.49, *p* > 0.05), and 18% to 9% during trip conditions (*t*(10)= −1, *p* > 0.05), the change was not significant. Similarly, the number of compensatory steps taken decreased pre- to post-training during slip conditions (*t*(10) = 2.20, *p* = 0.05) and pre- to post-training during trip conditions (*t*(10) = 1.74, *p* > 0.05); however, the change was not significant. Figure 1 represents the change in COM state stability, number of falls, and number of compensatory steps taken during the SPT test. Furthermore, all the individuals initiated a stepping response using their non-paretic limb pre- and post-training.

#### 3.1.2. Volitional Balance Control

**Figure 2:**
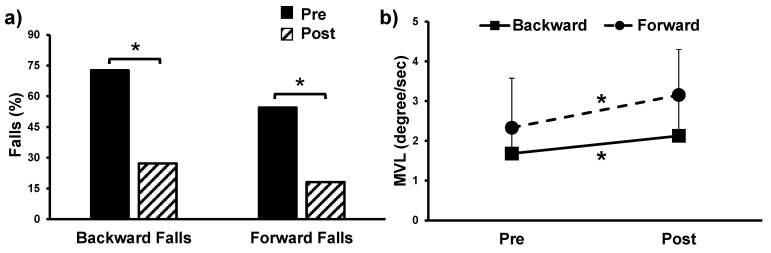
Change in pre-post training scores for participants on the (**a**) displays the significant change in fall percentage in backward and forward direction pre- to post-training in the Limits of Stability test (volitional balance) and (**b**) Significant main effect of time in the movement velocity (**a**) pre- to post-training in the Limits of Stability test (volitional balance). * *p* < 0.05.

Two by two ANOVA for MVL revealed a significant main effect of time (*F*(1, 20) = 6.958, *p* = 0.016) and group effect (*F*(1, 20) = 4.877, *p* = 0.039), indicating significant improvement on the LOS test post-intervention. However, there was no significant group by time effect (*F*(1, 20) = 0.616, *p* = 0.442) for MVL. Post-training number of falls recorded significantly decreased from 72% to 27% (*t*(10) = −2.88, *p* = 0.01) in the backward direction and 54.54% to 18.18% (*t*(10) = −2.39, *p* = 0.03) in the forward direction. Additionally, a large effect size was observed for backward sway (0.68) and forward sway (8.0). Figure 2 shows the increase in MVL and decrease in number of falls on the LOS test pre-post intervention.

#### 3.1.3. Clinical Balance Tests

**Figure 3:**
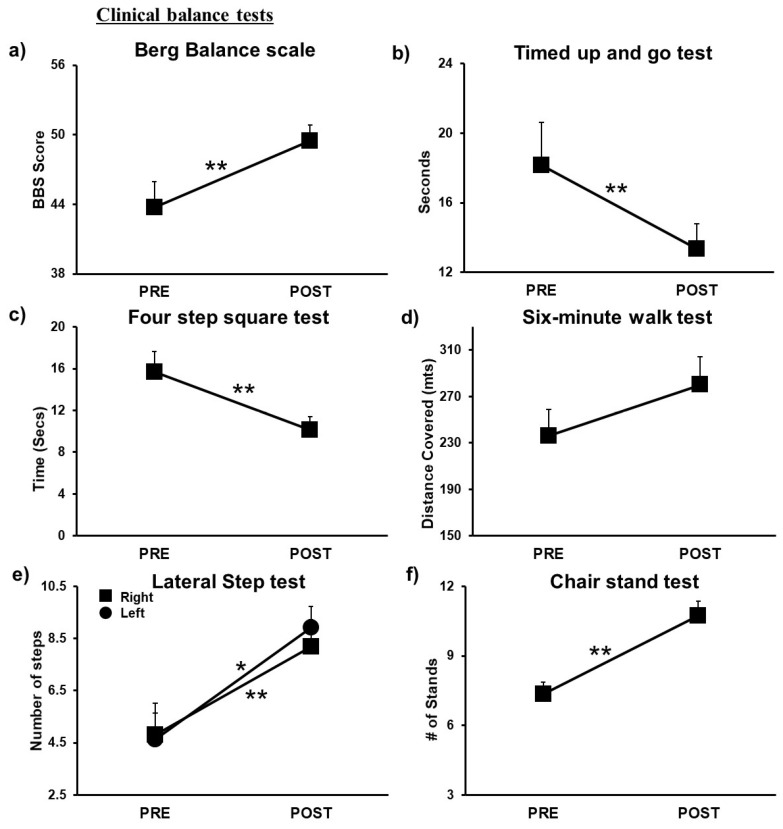
Post-training, individuals showed significant (**a**) increase in the Berg Balance Scale score, (**b**) reduced time to complete Timed Up-and-Go test (**c**) reduced time to complete Four Square Step Test, (**d**) increased distance for Six-Minute Walk test, (**e**) increased number of steps on the Lateral Time Step test, and (**f**) increased number of stands on Chair Stand test. * *p* < 0.05 ** *p* < 0.01.

A significant improvement pre-to post-intervention was observed in BBS with (*t*(10) = −3.84, *p* = 0.003) pre- to post-intervention with a large effect size (2.1) (Figure 3a). Similarly, time taken to complete the TUG (*t*(10) = 3.91, *p* = 0.003) pre- to post-intervention with large effect size (1.4) (Figure 3b), and FSST (*t*(10) = 5.785, *p* < 0.001) pre- to post-intervention significantly decreased with a large effect size (2.25) indicating better performance (Figure 3c). For the 6-MWT, a significant increase in total distance covered (*t*(10) = −3.19, *p* = 0.01) pre- to post-intervention with a large effect size (14.26) was observed (Figure 3d). Lastly, a significant increase in LST score with both left leg (*t*(10) = −3.45, *p* = 0.006) and right leg (*t*(10) = −2.734, *p* = 0.021) with a large effect size (6.5 and 6.24) (Figure 3e), and CST (*t*(10) = −4.39, *p* = 0.001) pre- to post-intervention with a large effect size (7.30) was observed (Figure 3f).

#### 3.1.4. Relationship between Laboratory Measures and Clinical Measures

Overall, it was found that change in laboratory measures (i.e., slip stability (*R*^2^ = 0.93, *p* > 0.05), trip stability (*R*^2^ = 0.58, *p* > 0.05), MVL backward (*R*^2^ = 0.38, *p* > 0.05) and MVL forward (*R*^2^ = 0.49, *p* > 0.05)) could not be predicted by change observed in clinical measures. The individual influence (beta values) of each clinical outcome on each laboratory measures is reported in Table 2.

## 4. Discussion

This study examined the effect of a six-week, multi-component, exercise-based conventional training intervention for improving both reactive and volitional balance control in PwCS. The results of this study support our hypothesis such that exercise-based balance training based on volitional movements showed limited benefits in improving postural stability on the reactive SPT. However, significant improvements were observed on the volitional LOS test and performance-based clinical balance tests.

### 4.1. Effect of Exercise-Based Conventional Training on Reactive Balance Control

In line with our hypothesis, the proposed EBCT implemented in this study showed limited improvements in reactive balance measured by COM state stability and number of falls on the SPT. This is contrary to previous studies that has shown improvement in reactive stepping measures and postural stability against stance perturbations [33,34]. However, these studies focused more on specific training to improve step reaction time [33,34]. Further, one of these studies included a mild form of reactive balance training by delivering unexpected manual pull-push [33]. Additionally, the reactive balance tests implemented in Marigold et al. 2005 and Vearrier et al. 2005 studies consisted of small magnitude stance perturbations (with lower accelerations and displacements) compared to our study [33,34].

In the current study, we examined reactive balance control on the SPT using kinematic parameters to induce a larger magnitude perturbation, mimicking more realistic environmental disturbances that might be experienced in the community. In this regard, both Vearrier 2005 [34] and Marigold 2005 [33] observed improvements in reactive balance control on time to stabilization of the center of pressure and latency of postural reflexes respectively in response to small magnitude perturbations, which did not elicit stepping responses; an important component to examine when assessing fall-resisting skills. Along this line, it has been well described that fixed support reactions, which maintain balance without changing the base of support, can be useful for maintaining balance against small perturbations [17,68]. However, it is change-in-support reactions that involve rapid stepping and grasping movements, and these are ultimately essential to re-establish balance and prevent falling [13,46,69,70]. Comparing our results with the previous study findings, it is possible to infer that EBCT based on volitional movements may help promote reactive balance control measures against small magnitude perturbations but not large magnitude perturbations. In line with this, as expected, the slip and/or trip stability did not have a positive correlation with any of the laboratory measures, further indicating the need to include assessment of reactive balance control in clinical settings as well.

### 4.2. Effect of Exercise-Based Conventional Training on Volitional Balance Control

In concordance with previous literatures, this study confirmed that EBCT improves volitional balance control assessed by LOS and clinical balance tests [23,24,25,26,27,28,29,30,31]. Several review studies focusing on multimodal training consisting of static and dynamic balance activities, strengthening and aerobic exercises, and gait training show improvements on the LOS and clinical balance tests similar to our study [28,29,71,72]. The positive effect of EBCT on volitional balance control observed in our study could be attributed to the task-specific nature of self-initiated and performance-based exercises which were repeatedly practiced for six weeks. In this regard, it is known that repeated practice of task promotes skilled motor performance and facilitates functional abilities when compared to non-repetitive training methods [73,74,75,76]. Most of the exercises included in our training protocol, such as standing on unstable surfaces (foam or tilt board), multidirectional reach-outs, stepping up to different heights, and forward and side lunges using both paretic and non-paretic limbs incorporated and facilitated weight shifting and rapid transitions in the center of mass, challenging the participants limits of stability. Thus, the concept of task-specificity, as well as the overlapping tasks from the clinical balance tests used for assessment, can explain the improvement on the LOS test. Despite these results, no correlation was observed between LOS laboratory measures and clinical outcome measure, which was expected as clinical tests usually assess different domains of balance control and even value in their final scores other motor functions such as gait and sit to stand strategies. Specifically, LOS examine individuals’ ability to control (voluntarily) their center of gravity in stance position which differs from the wide range of motor strategies that are evaluated in clinical tests such as BBS, TUG, 6MWT an d FSST.

Although several studies have established the benefit of task-specific perturbation training, it has been also demonstrated that, in neurophysiological terms, feedback responses (such as compensatory step after a postural disturbance) are clearly modulated throughout movement and are task-dependent [77,78,79,80]. In addition, it is shown that perturbations trigger reactive feedback responses that approximate in direction and magnitude followed by task-dependent voluntary responses [81]. This suggests that similar neural circuits may underlie both reactive and voluntary control systems, blurring the distinction between them [82]. This neurophysiological approach could support the idea that EBCT including voluntary movement related with reactive movement can enhance reactive balance behavior and reduce fall incidence. While the evidence shows significant and continued positive effects of EBCT in relation to dynamic stability and functional mobility in PwCS [25,26,27,28], it has been reported that the majority of ambulatory community-dwelling PwCS discharged from inpatient and outpatient rehabilitation continue to demonstrate deficits in reactive balance control and are unable to successfully use reactive stepping to recover balance loss following laboratory-induced perturbations [45,46,83,84]. The limited generalization of EBCT in improving reactive balance control, assuming a shared control mechanism between volitional and reactive stepping, probably stems due to other possible differences in neurophysiological mechanisms responsible for initiating and controlling the volitional and reactive balance domains.

### 4.3. Neurophysiological Differences between Voluntary and Reactive Balance Control

Neurophysiological studies have established that volitional balance control relies on feedforward mechanisms while reactive balance control relies on feedback mechanisms predominantly for balance recovery [13,14,15,16,85,86]. Volitional movements generate self-induced perturbations disturbing postural stability. To maintain postural stability and continue performing the volitional movements, the Central Nervous System (CNS) activates cortical and sub-cortical structures to program necessary actions which include activation of postural muscles and planning, sequencing, and activation of prime movers to generate the desired motor response [87,88,89,90]. On the other hand, in response to large-scale external perturbations, the CNS uses an internal representation of one’s stability limits to detect and determine the severity of balance loss and triggers appropriate stepping or grasping (or both) responses. It is postulated that these triggered responses involve supraspinal structures and descending pathways (long loop latency) with ongoing modulation of the response via the cerebellar-cortical loop [17,91,92,93,94,95].

In addition, several authors have established that initial compensatory responses do not correspond to changes in the perturbation-evoked cortical potentials that represent the sensory processing of the balance disturbance [96,97,98]. The onset latency of the afferent perturbation-evoked cortical potential is only slightly shorter than the initial muscle response and, therefore, the efferent path of the initial postural response is not properly timed with the afferent cortical potential in order to signify a cerebellar-cortical loop [97,98]. Similarly, another line of evidence suggests that a direct cerebellar-cortical loop does not trigger the initial phase of postural responses to external perturbations, but it seems likely that cerebral cortex become involved in later phases of the response [94,99]. According to these findings, therapeutic strategies based on volitional movements (exercise-based conventional balance training) might not have an effect on the earliest phases of reactive balance responses, given these earlier responses could represent peripheral sensory input mediated triggered synergies that are pre-set in the brainstem [94]. These neurophysiologic evidences could explain the different effect of EBCT on reactive and voluntary balance control. This also explains the no correlation observed between SPT and clinical tests outcome measures.

The study findings must be interpreted in light of its limitations. First, this is a pilot study and consisted of a limited sample size, which could have affected some results, such as the trend of decreasing in fall after the training observed in our results and/or the correlation between laboratory and clinical outcome measures. Nonetheless, there were large to very large effect sizes observed for most of the outcome measures, providing some confidence in the strength of the results. Second, the study only focused on biomechanical constraints of reactive balance control and intervention changes, if any, on neuromuscular responses need to be examined. Third, no control group was contemplated in the research design which could decrease the relevance of the results. Although the results of this study showed that exercise-based balance training based on volitional movements improves performance on clinical balance and LOS tests, and limited benefits in the SPT test, lack of control group could limit the interpretation of these results. The inclusion of a control group could have provided a perspective associated with the real effect of the experimental protocol in the current study such as a routine of self-care and physical exercise, or simply no intervention. However, even considering this limitation, the results presented in this study describe how a therapeutic protocol based on voluntary movements influences voluntary and reactive components of the balance control, which in turn contributes to the discussion associated with the specificity of the tasks required to train balance in populations at high risk of experiencing falls. Further, the purpose of this study was not to compare the proposed intervention in different groups, but to examine whether the intervention protocol implemented in this study impact on two different domains of balance control (volitional and reactive balance control). Lastly, the study was conducted in PwCS, so it remains to be determined if similar results would be obtained for people in sub-acute stages.

## 5. Conclusions

This study evaluated the effectiveness of a six-week, multi-component, exercise-based conventional training on volitional and reactive balance control in persons with stroke. The results indicate that exercise-based conventional training may have limited benefits in improving reactive balance control and fall-risk to external perturbations. However, it can significantly improve volitional balance control. While volitional and reactive balance control are both important for preventing falls, exercise-based conventional training may be combined with alternate training methods like perturbation training which may yield greater improvement in fall resisting skills among people with chronic stroke.

## Figures and Tables

**Table 1:** Characteristics of participants in the stroke group.

	Stroke (*n* = 11)
Age (years)	60.63 ± 4.24
Sex (Male/Female)	6/5
Site of Lesion (Left/Right)	6/5
Type of Stroke (Ischemic/Hemorrhagic)	4/7
Chronicity (years since stroke)	9.63 ± 6.63
CMSA- (Leg/Foot impairment)	4 ± 0.93/2.81 ± 1.53

CMSA: Chedoke–McMaster Stroke Assessment.

**Table 2:** Beta values of linear regression to understand the relationship between laboratory and clinical measures.

	Slip Stability	Trip Stability	LOS Backward	LOS Forward
Berg balance scale	−0.01	−0.005	−0.145	−0.045
timed up and go test	0.005	−0.003	−0.44	−0.376
6-min walk test	−0.001	−0.001	−0.010	0.011
Lateral step test	0.38	0.042	0.076	0.326
Chair stand test	−0.36	−0.07	0.433	−0.658
Four step square test	−0.036	−0.074	0.105	0.223

LOS: Limits of stability.
